# Hydration-Induced Phase Separation in Amphiphilic Polymer Matrices and its Influence on Voclosporin Release

**DOI:** 10.3390/jfb3040745

**Published:** 2012-10-30

**Authors:** I. John Khan, N. Sanjeeva Murthy, Joachim Kohn

**Affiliations:** New Jersey Center for Biomaterials, the State University of New Jersey, Piscataway, NJ 08854, USA; Email: johnkhan@verizon.net (I.J.K.); murthy@biology.rutgers.edu (N.S.M.)

**Keywords:** hydration-induced phase separation, amphiphilic polymer, resorption, voclosporin, small angle scattering

## Abstract

Voclosporin is a highly potent, new cyclosporine-A derivative that is currently in Phase 3 clinical trials in the USA as a potential treatment for inflammatory diseases of the eye. Voclosporin represents a number of very sparingly soluble drugs that are difficult to administer. We therefore selected it as a model drug that is dispersed within amphiphilic polymer matrices, and investigated the changing morphology of the matrices using neutron and x-ray scattering during voclosporin release and polymer resorption. The hydrophobic segments of the amphiphilic polymer chain are comprised of desaminotyrosyl-tyrosine ethyl ester (DTE) and desaminotyrosyl-tyrosine (DT), and the hydrophilic component is poly(ethylene glycol) (PEG). Water uptake in these matrices resulted in the phase separation of hydrophobic and hydrophilic domains that are a few hundred Angstroms apart. These water-driven morphological changes influenced the release profile of voclosporin and facilitated a burst-free release from the polymer. No such morphological reorganization was observed in poly(lactide-*co*-glycolide) (PLGA), which exhibits an extended lag period, followed by a burst-like release of voclosporin when the polymer was degraded. An understanding of the effect of polymer composition on the hydration behavior is central to understanding and controlling the phase behavior and resorption characteristics of the matrix for achieving long-term controlled release of hydrophobic drugs such as voclosporin.

## 1. Introduction

Biodegradable polymers are widely used as matrices for drug delivery with different methods of formulation pursued for improving the delivery of a variety of hydrophobic small molecules and peptides. The rate of release of these active ingredients is determined by their interactions with the matrix and by the degradation and resorption of the matrix, which in turn are influenced by water uptake and its distribution [1,2]. Biodegradable, hydrophobic polymers are useful as drug-release matrices because they maintain structural integrity while eroding slowly. These polymers can also be copolymerized with a hydrophilic polymer, often poly(ethylene glycol) (PEG), to control the hydration behavior and thus affect the release of active ingredients.

Few studies in the literature address the effect of morphology of hydrated drug-loaded polymer matrices on degradation, erosion and drug release [3]. Water is known to exist in different states (bound and free water) and may form clusters depending on the ratio of hydrophobic and hydrophilic domains in polymers [4]. The structure [5,6] and the hydration [7,8,9] of PEG-containing copolymers, and the clustering of water in polymers [4,10,11,12,13] are expected to affect both drug release and polymer resorption. For example, water is shown to disrupt the crystalline nature of PEG incorporated into hydrophobic chains of either poly(tetramethylene oxide) and poly(dimethyl siloxane) [14]. Such disruption is due to the interactions between water molecules and polymer chains, which sometimes result in phase separation of polymer domains. Our study indicates that the distribution of hydrophobic-hydrophilic segments can affect the bulk polymer morphology and that water molecules can play an important role in influencing this distribution, and subsequent polymer degradation.

In the work presented here, we describe how the chemical composition and morphology of our polymer matrix modulates the release of a hydrophobic undecapeptide (voclosporin). We selected the polymers from a library of biodegradable random copolymers comprised of PEG and derivatives of tyrosine dipeptide. These tyrosine-derived polymers have excellent biocompatibility and find utility in many biomedical applications [15,16,17]. Voclosporin is a new drug, similar to cyclosporine-A. It is currently in clinical trials for indications in ophthalmic inflammatory diseases such as dry eye syndrome, uveitis and blepharitis [18]. Like cyclosporine-A, voclosporin is a hydrophobic peptide that can serve as a model for a large number of drug candidates that exhibit low solubility and poor bioavailability when administered.

The significance of this study is to address the inadequate release profile of voclosporin (and other hydrophobic drugs) from commonly used poly(lactide-*co*-glycolide) (PLGA) matrices. These release profiles are characterized by long initial lag periods (no drug release) followed by drug burst when the polymer matrices begin to resorb. These lag-burst release profiles are not usually clinically relevant. In contrast, we observe a sustained release of voclosporin without lag or burst effects from hydrophilic-hydrophobic copolymers consisting of desaminotyrosyl-tyrosine ethyl ester (DTE), desaminotyrosyl-tyrosine (DT) and small segments of PEG. We therefore investigate the changes in morphology during hydration in these copolymer matrices, and their effect on drug diffusion, using wide-angle X-ray scattering (WAXS), small-angle x-ray scattering (SAXS) and small-angle neutron scattering (SANS). WAXS provides information at 1–10 Å length scales (molecular structures). SAXS provides information about the microphase separation at 100–1000 Å length scales (meso-length scale) by making use of the contrast in electron densities between the different phases of the polymer. SANS provides information about the distribution of water, at the same length scales as SAXS, when polymers hydrated with deuterated water generate contrast [19]. These techniques are ideally suited for characterizing the changes in bulk morphology during hydration and aging in polymers containing hydrophilic segments such as PEG. The results are useful for developing a structural basis that can explain the release characteristics of voclosporin (and other hydrophobic drugs) from amphiphilic copolymers.

## 2. Results and Discussion

### 2.1. Nomenclature for Tyrosine-derived Polymer Matrices

A shorthand notation for tyrosine-derived copolymers, Eyyzz, is used where “E” refers to DTE, “yy” is the mole% of DT, and “zz” is the mole% of PEG_1K_. For example, E1218 refers to a copolymer with 12 mole% of DT, 18 mole% of PEG_1K_ and 70 mole% of DTE (implied since the molar fractions of all three components must add up to 100%). 

### 2.2. Voclosporin Release is Dependent on Matrix Composition

**Figure 1:**
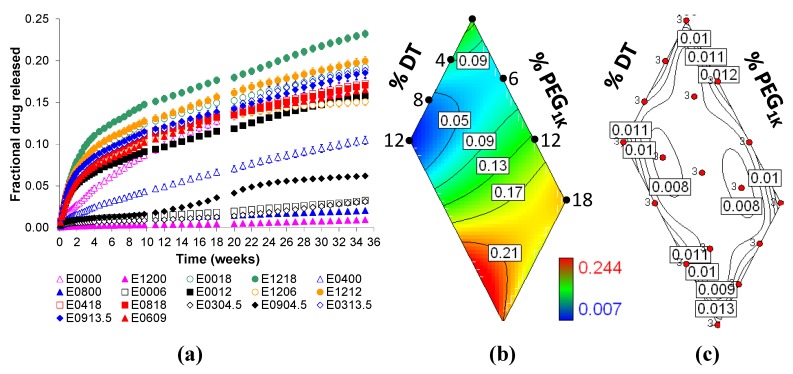
The influence of molar fractions of desaminotyrosyl-tyrosine (DT) and poly(ethylene glycol) (PEG_1K_) in Eyyzz matrices on *in vitro* cumulative fractional release of voclosporin: (**a**) release profiles up to 35 weeks; (**b**) contour plot of release at 35 weeks; and (**c**) contour plot for 35-week standard error (in box), where red dots indicate polymer composition design points and ‘3’ represents the sample size (see text for additional detail).

The *in vitro* cumulative fractional release of voclosporin (initial loading of 30 wt.%) over a 35-week period was dependent on the relative amounts of DTE, DT and PEG_1K_ present in the polymer (Figure 1a). There is a gradual change in the amount of voclosporin released from the various matrix compositions. Figure 1b illustrates three trends of how voclosporin release is controlled by the matrix composition. In this figure, blue (contour line < 0.05) and red (contour line > 0.21) regions indicate the lowest and highest amount of fractional release, respectively. First, there is a reduction in release as the DT content increases in Eyy00 (left edge labeled as %DT). Second, there is an increase in release as PEG content increases in E00zz (right edge labeled as %PEG_1K_). Third, there is a synergistic effect of DT and PEG components (red region) that further increases voclosporin release in Eyyzz. The synergy between DT and PEG is a surprising and so far unprecedented observation for which we have currently no obvious explanation. The contour map therefore enables the design of the release matrices: the path from contours of low to high cumulative release (0.09 to 0.21 in the figure) leads towards the composition with a faster release of voclosporin.

### 2.3. PLGA is Unsuitable for Controlled Voclosporin Release

**Figure 2:**
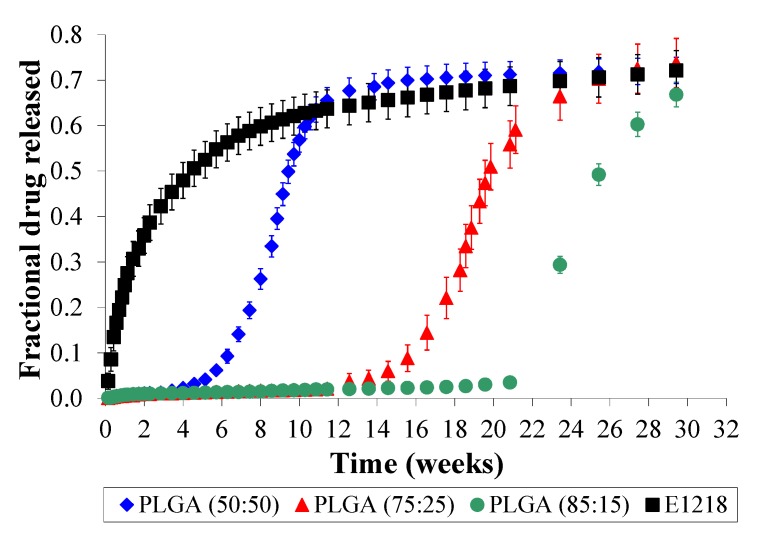
*In vitro* lag and burst release profiles of voclosporin (5 wt.% loading) is observed only in poly(lactide-*co*-glycolide) (PLGA) compositions (50:50, 75:25 and 85:15), and not in Eyyzz copolymers (illustrated using E1218 release profile).

Considering the frequent use of PLGA as a drug release matrix, we used three different PLGA compositions (50:50, 75:25 and 85:15) to compare the voclosporin release profiles obtained from these polymers to those obtained from the phase-separated, amphiphilic polymers, exemplified here by E1218. In order to obtain higher cumulative fractional voclosporin release from the matrices, we used an initial voclosporin loading of 5 wt.%. The data (Figure 2) from the three PLGA compositions 50:50, 75:25 and 85:15 show that there is a lag period (*i.e.*, no voclosporin release) of approximately 1, 3 and 5 months, respectively. This lag period is followed by a 6-week burst of approximately 50%–60% of the voclosporin, and a subsequent final slow release of the residual voclosporin. Similar sigmoidal profiles of drug release from PLGA are commonly reported in the literature [20]. Apparently, voclosporin and other highly hydrophobic molecules can remain trapped within the polymer until the matrix has significantly degraded. This associated lag period, followed by a strong burst release from the degrading matrix is rarely useful in clinical settings. In contrast, the 30-week *in vitro* release profile of voclosporin from E1218 is different. Voclosporin release starts immediately upon immersion in PBS and continues without lag or burst throughout the entire 30-week period. During this time, 48 ± 10% of the voclosporin is released within the first 4 weeks, while 24 ± 2% of the voclosporin is eluted from the matrix within the subsequent 26 weeks. 

### 2.4. Bulk Polymer Resorption is Dependent on Matrix Composition

**Figure 3:**
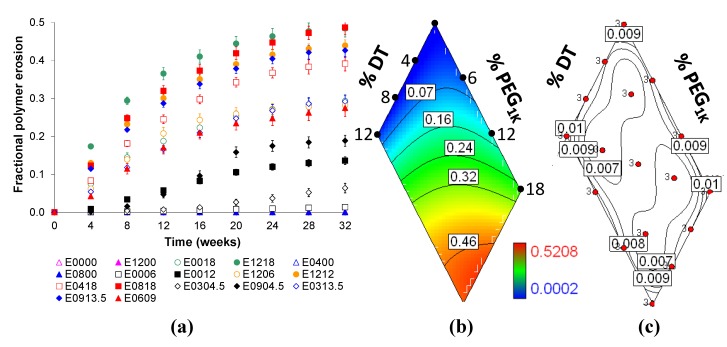
The influence of molar fractions of DT and PEG_1K_ in Eyyzz matrices on *in vitro* polymer resorption: (**a**) release profiles up to 32 weeks; (**b**) contour plot of release at 32 weeks, and (**c**) contour plot for 32-week standard error (in box), where red dots indicate polymer composition design points and “3” represents the sample size (see text for additional detail).

The *in vitro* cumulative fractional resorption of Eyyzz matrices loaded with voclosporin (30 wt.%) were characterized over a 32-week period. The amount of resorption is dependent on the molar fractions of DTE, DT and PEG_1K_ present in the matrices, and the results are presented in Figure 3a. Figure 3b illustrates trends of how the polymer is eroded. In this figure, blue (contour line < 0.07) and red (contour line > 0.46) regions in the contour map indicate the lowest and highest rate of polymer resorption, respectively. There is no significant change in resorption as the DT content increases in Eyy00 (left edge labeled as %DT). However, resorption did increase as the PEG content increases in E00zz (right edge labeled as %PEG_1K_). We again observe a surprising synergistic effect when both DT and PEG are present as evidenced by the further increase in resorption in Eyyzz (the red region, contour line > 0.46). As in the previous contour map, the trajectory from contours of low to high fractional resorption, 0.07 to 0.46 in the figure, leads towards a composition that attains faster polymer resorption during hydration.

**Figure 4:**
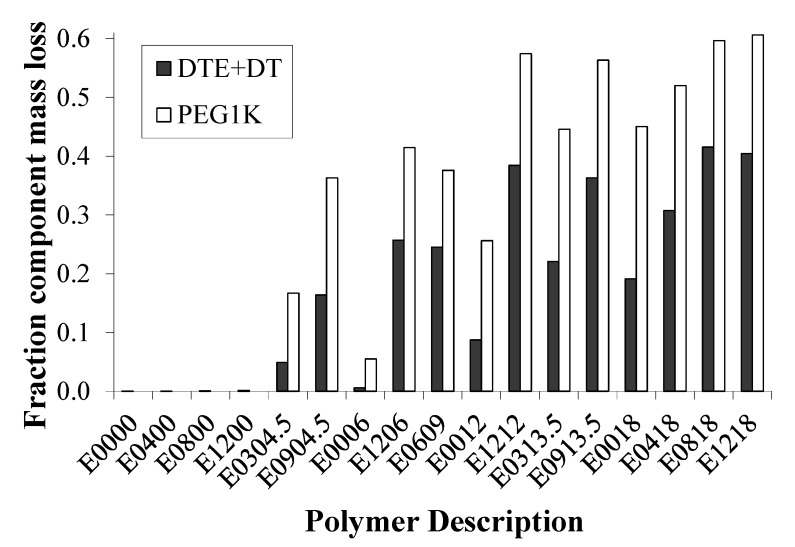
Cumulative fractional erosion of desaminotyrosyl-tyrosine ethyl ester (DTE) + DT and PEG_1K_ components of polymer matrices loaded with voclosporin (30 wt.%) demonstrate that PEG_1K_ segments are preferentially lost from the matrix relative to the hydrophobic segments, resulting in matrices that change in composition over time.

In order to determine the levels of hydrophobic (DTE + DT) and hydrophilic (PEG_1K_) components that were being eroded from the matrix during the study, we calculated their percentages and plotted the cumulative amounts of each after 32 weeks of resorption as shown in Figure 4. In general, the erosion of PEG segments is 1.4 to 3.4 times faster than the erosion of the DTE + DT segments. This preferential loss of PEG was confirmed by measuring the dry *T_g_* of E1218 and E0000, a relatively fast and relatively slow eroding matrix, respectively. During a 20-week incubation in PBS at 37 °C, the measured dry *T_g_* of E1218 increased from 7 °C to 15, 18, 24 and 29 °C after 1, 4, 10 and 20 weeks, respectively. We attributed the observed increase in the dry *T_g_* to the preferential loss of the flexible component (PEG) from the polymer composition. In contrast, the dry *T_g_* of E0000 remained constant at 95 °C during the same period. Additionally, there was an inverse linear relationship (R^2 ^= −0.975) between the dry *T_g_* (in °C) and the % PEG_1K_ content of the Eyyzz copolymers. The empirical equation for calculating the remaining PEG in a resorbed polymer is given below:

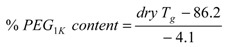
(1)
Using Equation 1, the amount of PEG in the 20-week hydrated E1218 is estimated at 14 mole%, based on the dry *T_g_* measurement stated above. Thus, although PEG is preferentially eroded from this matrix, there appears to be adequate amounts of PEG (and water) available. 

It should be noted that the weak correlation between PEG resorption and voclosporin release does not necessarily imply that there is a causal relationship between the two. 

### 2.5. State of Voclosporin in Polymer Matrices

Structural changes in the polymers at length scales of 2–15 Å (*q* range ~ 0.4–3.1 Å^−1^) were examined using WAXS. The hydrated polymers showed three amorphous peaks: one at *q* ~ 1.4 Å^−1^ due to an average spacing between neighboring polymer chains of ~ 4.5 Å, and at two additional peaks *q* ~ 1.9 and ~ 2.9 Å^−1^ due to short-range ordering of the PEG-water complex. Two new peaks at *q* ~ 0.6 and 1.2 Å^−1^ appeared in drug-loaded polymers at greater than 10 wt.% loading. The intensities of these peaks increased with drug loading, and were approximately at the same position as the most intense peaks of the drug crystals. This shows that the drug is amorphous within the polymer matrix, and remains in this state as the polymer erodes. In contrast, crystalline peaks of the drug began to appear in the PLGA matrices, and were fully developed after seven weeks of hydration. Drug burst began as the drug started to recrystallize from the matrix.

### 2.6. Water Domains are Formed in Eyyzz Copolymers

**Figure 5:**
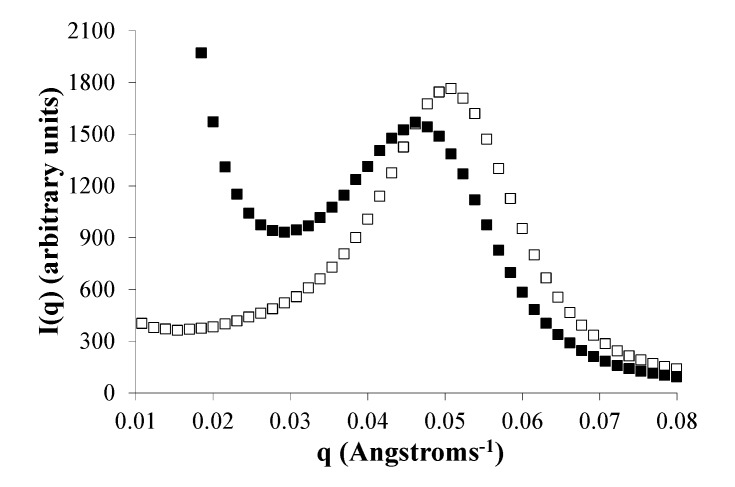
Typical small-angle neutron scattering (SANS) scans are illustrated using E1218 with voclosporin (filled square) and without voclosporin (open square) after incubation in deuterated phosphate buffered saline (PBS) for 60 hours. Central diffuse scattering (CDS) occurs only in the voclosporin-loaded polymer for *q* < 0.015 Å, and the interference peak is shifted to the left (lower *q* value means larger water domain spacing).

Small-angle neutron scattering scans from all dry polymers were featureless, whereas the hydrated polymers with > 6 mole% PEG_1K_ show an interference peak (Figure 5). This interference peak at *q_max_* of 0.052–0.029 Å^−1^, which is illustrated using E1218, signifies the formation of structured domains of water within these matrices. The distances between the domains in E1218 were estimated at 110–190 Å from curve fitting the data with the extended Zernike-Prins model, and water domain diameters were estimated in the range of 80–140 Å. Domain spacings and diameters were larger in polymers with larger PEG content (or large water uptake). In each hydrated polymer, the presence of voclosporin gave rise to intense central diffuse scattering (CDS, see next paragraph), but there was no substantial difference in either the measured water domain size or the domain spacing.

A second feature in the SANS data, seen only in E00zz copolymers with voclosporin, is the intense scattering at low *q* in the range of 0.008–0.018 Å^−1^. This was observed in SAXS data as well. We attribute this additional intensity (*i.e.*, CDS) to inhomogeneous distribution of the hydrated domains, most likely to the clustering of hydrated domains in the presence of the hydrophobic drug. CDS has been observed in triblock polymers containing PEG [21]. The diameters of the CDS domains in these matrices are 2–7 times larger than the diameters of water domains associated with the interference peak.

**Figure 6:**
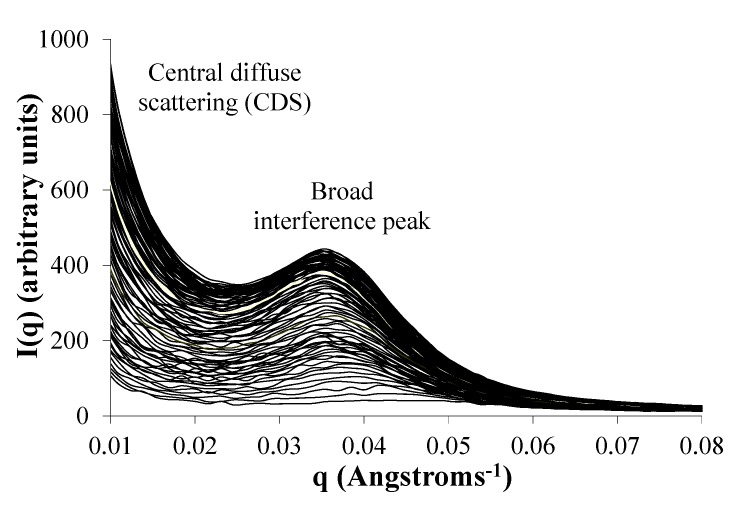
Water domain evolution in E1218 loaded with voclosporin. Each SANS scan (depicted as separate curves) was taken sequentially at time intervals of 30–120 seconds.

The evolution of water domains in these matrices from the beginning of water exposure was followed in one of the polymer formulations—the voclosporin-loaded E1218 (Figure 6). E1218 was chosen since it had relatively high amounts of both polymer erosion and voclosporin release. The interference peak and CDS are detectable within 120 seconds after contact with water, and both features continued to increase in intensity for up to 2 hours (as shown here), and beyond. The data show that there is rapid formation of structured domains of water due to the ingress and redistribution of water molecules. 

### 2.7. Phase Separation of PEG Segments Occur in Hydrated Eyyzz Copolymers

The interference peak in SANS data (Figure 5 and Figure 6) shows that water rapidly enters the polymer matrix forming clusters of water-rich domains. A similar peak was observed in SAXS data as well. This SAXS peak shows the formation of ordered PEG domains, whose size and separation are comparable to those of the water clusters. Similarities in the size and the spacing of the water domains in SANS and PEG domains in SAXS (Table 1) lead to the conclusion that the water-rich domains and PEG domains are the same. All hydrated Eyyzz matrices, with or without voclosporin, showed an interference peak that signified the presence of structured polymer domains. For each Eyyzz with PEG content ≥ 6 mole%, the peak position shifts to lower values as the hydration time of the matrix increases from 1 to 7 weeks (Table 1). We estimated the corresponding domain spacing (d_PEG_) and domain size (D_PEG_) by curve fitting the SAXS data using the extended Zernike-Prins model.

Ordered domains, evidenced by the presence of interference peaks, are commonly observed even in the dry state in di- and tri-block copolymers [22], but not in random copolymers. Furthermore, hydrated domains of PEG have been observed in a PEG-containing poly(ether ester amide), in which phase separation present in the dry state became more pronounced upon hydration [23]. In addition, a peak in the SAXS structure factor for hydrophobic-modified polyacrylamide gels indicated that hydrophobic interactions can promote nanophase separation [24]. As an extension of these observations, we report on the formation of hydrated ordered domains exclusively due to absorption of water in our PEG-containing random segmented amphiphilic copolymers. Enthalpic contribution from hydrogen bonding with water molecules could enable the mobile hydrated PEG segments to overcome the entropic free energy associated with the random distribution of PEG resulting in phase separation from the hydrophobic regions. This phase separation upon hydration is important in understanding the steady state drug release from Eyyzz polymers.

**Table 1:** Domain spacing (*d*_PEG_) and diameter (D_PEG_) determined from small-angle X-ray scattering (SAXS) data of dry and hydrated polymer matrices loaded with voclosporin.

Polymer composition	*d_PEG_* (Å), for polymer + VCS				*D_PEG_* (Å), for polymer + VCS			
	dry	1 wk	4 wk	7 wk	Dry	1 wk	4 wk	7 wk
E0000	-	-	-	-	-	-	-	-
E0400	247	283	425	291	204	183	234	229
E0800	291	440	-	331	234	224		229
E1200	274	446	464	414	234	302	276	280
E0304.5	-	-	323^(b)^	-	-	-	159^(b)^	-
E0904.5	-	-	321^(b)^	-	-	-	220^(b)^	-
E0006	-	212	213	264	-	132	172	234
E1206	-	137^(a)^	244		-	132^(a)^	203	-
E0609	-	166	214	304	-	125	166	264
E0012	-	194	246	333	-	137	159	222
E1212	-	133	157	297	-	124	183	305
E0313.5	-	149^(a)^	180	266	-	156^(a)^	178	253
E0913.5	-	138	148	256	-	118	159	258
E0018	-	133	154	228	-	163	176	225
E0418	-	137	143	210	-	128	156	213
E0818	-	121	135	201	-	124	145	209
E1218	-	101	132	204	-	121	159	213
E1818	-	^(c)^	^(c)^	192	-	^(c)^	^(c)^	197
E1420	-	99	^(c)^	179	-	93	^(c)^	178
E1224	-	93	^(c)^	175	-	105	^(c)^	179
E1230	-	81	^(c)^	133	-	80	^(c)^	134
DT homopolymer	-	-	-	-	-	-	-	-
PEG homopolymer	126	^(c)^	^(c)^	^(c)^	146	^(c)^	^(c)^	^(c)^

The absence of values indicates that no interference peak was detected: ^(a)^ Not used for PEG trend analysis due to appearance of split peaks; ^(b)^ Not used for PEG trend analysis since PEG content < 6 mole%; ^(c)^ Not able to test since sample was fragmented.

Table 1 shows, in general, that for each hydration time, the PEG domain spacing decreases as the PEG content increases (valid for compositions with PEG_1K_ ≥ 6 mole%), along with a concomitant small increase in domain size. In addition, across the hydration times ranging from 1 to 7 weeks, we observe that both the PEG domain size and spacing increases. Similar trends were obtained for PEG-containing matrices that did not contain voclosporin.

Additional examination of the data reveals that dry polymers are featureless, with the exception of Eyy00 copolymers loaded with voclosporin that manifested an interference peak. These copolymers did not show an interference peak in the absence of voclosporin. The presence of the peak indicates a potential structured complex between the voclosporin and the DTE*-co-*DT segments of the polymer. This peak persisted during hydration, shifting to lower *q_max_* during the 7-week period. It is important to notice that voclosporin loaded in either E0000 or DT homopolymer did not give rise to any measurable SAXS signal. In addition, there was no measurable diffraction pattern in any hydrated PLGA samples as these matrices were homogeneous and did not form phase separated domains as found in the Eyyzz. Finally, the interference peak in the dry homopolymer of PEG, present in samples with and without voclosporin, is due to the well-known folded-chain lamellar structure of this polymer [25].

These morphological changes in Eyyzz, which are driven by water and are dependent on the polymer composition, account for the controlled release of hydrophobic drugs such as voclosporin. This behavior is not observed in PLGA.

## 3. Experimental Section

### 3.1. Active Pharmaceutical Ingredient

Voclosporin was a gift from Lux Biosciences Inc. (Jersey City, NJ, USA).

### 3.2. Synthesis of Tyrosine-Derived Polymer Matrices

The monomers, DTE and desaminotyrosyl-tyrosine tert-butyl ester (DTtBu), were synthesized as described in previous publications [17]. Tyrosine-derived polycarbonates were synthesized at room temperature via a condensation reaction of pyridine, triphosgene and up to three monomers—DTE, DTtBu and PEG—depending on the desired composition [17]. Polymers containing DTtBu were deprotected using trifluoroacetic acid (TFA) forming DT units. All polymers were washed with a 1:1 (vol./vol.) mixture of isopropyl alcohol and methanol, and filtered after dissolving in dichloromethane.

### 3.3. Poly(DL-Lactide-co-Glycolide), PLGA

PLGA 50:50 (molecular weight 40–75 kDa), PLGA 75:25 (molecular weight 66–107 kDa) and PLGA 85:15 (molecular weight 50–75 kDa), purchased from Sigma-Aldrich Chemicals Inc. (St. Louis, MO, USA), were used as received.

### 3.4. Sample Preparation

Preforms of drug-loaded matrices were prepared by solvent casting from dichloromethane, followed by drying under nitrogen at room temperature. Thin films with uniform thicknesses were made from the preformed material by 5 minutes of hot compression molding at temperatures of 30–50 °C above the glass transition temperature (*T_g_*) of the matrix [26,27]. Control film samples were prepared in a similar manner without voclosporin.

### 3.5. *In Vitro* Kinetic Drug Release (KDR) Study

Voclosporin-loaded polymers were immersed in phosphate buffered saline (PBS) at pH 7.4 and 37 °C. At each time point, the buffer was removed for quantitative analysis and replaced with a fresh solution for subsequent time points, where the volume was adjusted as necessary to maintain sink conditions throughout the study.

Quantitative analysis was performed by high performance liquid chromatography (HPLC) with a linear gradient of mobile phase A comprised of water with 0.1% TFA and mobile phase B comprised of acetonitrile with 0.1% TFA (0 min: 30% A and 70% B; 7.5 min: 25% A and 75% B). A C18 column (33 × 4.6 mm; 3 μm particle size) was run at 60 °C at a flow rate of 0.8 mL/min. Voclosporin was detected at 210 nm. The limits of quantification and detection for the HPLC setup were 25 and 6.3 ng/ml, respectively. The sample size for each formulation was 3.

### 3.6. *In Vitro* Polymer Erosion Study

Voclosporin-loaded polymers were incubated in buffer as described above. Samples were prepared for quantitative analysis by degrading the dissolved polymer with sodium hydroxide. The base treatment converted all DTE into DT, and cleaved the solubilized polymer backbone into DT monomers and PEG_1K_ fragments. The destruction of voclosporin using this method was not relevant for the analysis.

Quantitative analysis was performed by HPLC with an in-line evaporative light scattering detector (ELSD). A linear gradient of A and B was used (0 min: 95% A and 5% B; 15 min: 60% A and 40% B; 16 min: 50% A and 50% B; 17 min: 5% A and 95% B; 20 min: 5% A and 95% B; 21 min: 95% A and 5% B). A C18 column (33 × 4.6 mm; 3 μm particle size) was run at 25 °C at a flow rate of 0.8 mL/min. The DT monomer was detected at 220 nm and the PEG_1K_ was detected with the ELSD. The sample size for each formulation was 3.

### 3.7. *In Vitro* Experimental Mixture Design for KDR and Polymer Erosion Studies

Response surface methodology was used to select Eyyzz compositions in a d-optimal design space using Design Expert® software version 7.1.6 (Stat-Ease Inc., Minneapolis, MN). The compositional limits of “yy” = 12 mole% and “zz” = 18 mole% were selected after preliminary studies showed that sample fragmentation occurred at higher compositional limits with samples containing 30% (wt./wt.) of voclosporin. The general form of the reduced-cubic polynomial used to fit the data was [28]:

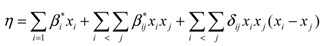
(2)
where *η* is the response variable (*i.e.*, voclosporin release or polymer resorption); *x_i_*, *x_j_* and *x_k_* are the mixture component terms with *i*, *j* and *k* representing the three monomers DTE, DT and PEG_1K_; and β_i_*, β_ij_* and *δ_ij_* are the partial regression coefficients of the polynomial model. Estimation of the regression coefficients was via the method of least squares, and analysis of variance was used to select statistically significant terms in the model. Contour plots describe the empirical relationship between the response variable and the polymer composition.

### 3.8. Measurement of Changes in T_g_ During Polymer Erosion

Two polymers, E0000 and E1218, were tested without voclosporin. These samples were immersed in PBS at pH 7.4 and 37 °C with weekly changes of the buffer. At each time point, the hydrated polymer was dried by lyophilization and the dry *T_g_* was measured by differential scanning calorimetry. The sample was heated from –50 to 200 °C at a rate of 10 °C per minute, kept at 200 °C for 5 minutes to erase the thermal history of the polymer (first heat cycle), cooled, and then reheated from –50 °C to 200 °C at a rate of 10 °C per minute (second heat cycle). The sample size for each polymer was 3.

### 3.9. SANS Measurements

Test samples (~ 0.2 mm thick) of polymer-only or voclosporin-loaded polymers were incubated in deuterated PBS at pH 7.4 and 37 °C. SANS data were collected at the CG-2 beam line (Oak Ridge National Laboratories, Oak Ridge, TN) at a wavelength of 4.766 Å and a sample-to-detector distance of 8.65 meters over a *q* range of 0.007–0.154 Å^−1^. Data collection time varied from 20 to 1200 seconds.

### 3.10. SAXS and WAXS Measurements

Test samples (~ 1 mm thick) of polymer-only or voclosporin-loaded polymers were incubated in PBS at pH 7.4 and 37 °C for 1, 4 and 7 weeks. X-ray scattering data were collected on the 5ID-D beam line at the Advanced Photon Source (APS, Argonne National Laboratory, Argonne, IL, USA) using 1.0332 Å radiation and a beam size of 0.2 × 0.2 mm in transmission mode at room temperature. WAXS data were collected on a 100 × 200 mm CCD camera (NTE/CCD-1340/1300E CCD module, Princeton Instruments, Inc., Trenton, NJ) with a sample-to-detector distance of 0.23 m over a *q* range of 0.43–3.21 Å^−1^. SAXS data were collected on a 100 × 100 mm CCD camera (NTE/CCD-1340/1300E CCD module, Princeton Instruments, Inc., Trenton, NJ, USA) with a sample-to-detector distance of 2.897 m and *q* range of 0.006–0.160 Å^−1^. Dark frame, distortion and flat-field corrections were performed using FIT2D software [29]. Exposure time per sample for data collection ranged from 0.1–12 seconds. Scattering from an empty cell was collected for background subtraction.

### 3.11. Analysis of Small Angle Scattering Data

The SANS and SAXS data were fitted with an extended Zernike–Prins model. This model is based on a one-dimensional arrangement of spherical scatterers [30]. This simple model was adequate for determining the size and spacing of water or PEG domains in hydrated polymers. The extension of the Zernike–Prins model was the addition of a Guinier approximation term of the form [*I_0_^’^ exp(-q^2^R_g_^2^/3)*] to take into account a decaying exponential signal at very low *q* values (< 0.015 Å^−1^). This term described the CDS from a monodisperse system of spherical particles [31]. The extended ZP model is given by:

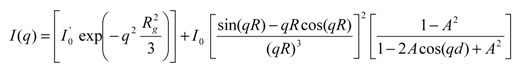
(3)
where, *q* is scattering vector; *I(q)* is the measured scattering intensity; *I_0_^’^* and *I_0_* are the intensities at *q* = 0 for CDS and the interference peaks, respectively; *R_g_* is the radius of gyration of the CDS domains; *R* and *d* are the radius and interdomain spacing of hydrated PEG domains, respectively; *A* = *exp(−q^2^σ^2^/2)*, where *σ* is the distribution of the interdomain spacing of water or PEG domains.

Scattering curves were simulated by coding Equation 3 in Microsoft Excel^®^ Solver with *I_0_^’^*, *I_0_*, *R_g_*, *R*, *d* and *σ* as the variable parameters. Observed curves were profile fitted by minimizing the root mean square difference between the computed and the experimental intensities. The fit was performed in the *q* range of the peak of interest. Initial values for these parameters were iterated until a best fit was obtained.

## 4. Conclusions

The amphiphilic Eyyzz copolymers exhibit a unique advantage as a release matrix over the commonly useful hydrophobic biomaterial PLGA. PLGA is unsuitable due to its voclosporin retention and ensuing burst release. In contrast, a long-term controlled release of voclosporin was evident in Eyyzz having PEG_1K_ content > 6 mole%. The benefit of these Eyyzz compositions originates from their dynamic nature: water uptake creates phase separation of its hydrophobic and hydrophilic segments, and resorption slowly changes the composition of the polymer. We propose that phase separation might sequester voclosporin within the hydrophobic polymer domains and prevent its burst release. Phase separation was demonstrated by SANS and SAXS measurements of the hydrated polymer. SANS measurements showed rapid uptake of water that forms clusters of water-rich domains. SAXS measurements showed the formation of ordered PEG domains, whose size and separation are comparable to those of the water clusters. The similarities in structure development and dimensions imply that the water-rich domains and PEG domains are the same.
